# Can touch this: training to correct police officer beliefs about overdose from incidental contact with fentanyl

**DOI:** 10.1186/s40352-021-00163-5

**Published:** 2021-11-24

**Authors:** Brandon del Pozo, Emily Sightes, Sunyou Kang, Jeremiah Goulka, Bradley Ray, Leo A. Beletsky

**Affiliations:** 1 The Miriam Hospital/Warren Alpert Medical School of Brown University, 164 Summit Ave, Providence, RI 02906 USA; 2 Center for Behavioral Health and Justice, Wayne State University School of Social Work, Detroit, USA; 3 Health in Justice Action Lab, Northeastern University School of Law, Boston, USA; 4 Northeastern University Bouve College of Health Sciences, Boston, USA

**Keywords:** Police, Law enforcement, Overdose, Stigma, Opioids, Fentanyl

## Abstract

Misinformation about overdose risk from accidentally inhaling or touching fentanyl is widespread among police in the United States. This may aggravate already elevated burdens of officer stress and burnout, while chilling lifesaving overdose response. Police education has shown promise in reducing false beliefs about fentanyl. To better understand the potential of training interventions in correcting officer knowledge, we administered a 10-min online training with corrective messaging about occupational overdose risk from fentanyl contact to 204 police officers in Indiana. Overall, 129 officers (63%) completed baseline survey and 69 (34%) completed follow-up instrument. Using a 6-point Likert scale, we documented assent with the statement: “First responders who encounter fentanyl are at great risk of overdose by touching it or inhaling it.” At baseline, 79.8% expressed agreement, while 20.2% disagreed. At follow-up, 39.1% agreed, while 60.9% disagreed (*p* < .001). Baseline responses varied in that those officers without a college degree and those on patrol were more likely to report false beliefs. A brief online training intervention holds promise for correcting false beliefs about the risk of fentanyl overdose under circumstances commonly encountered by police.

## Introduction

In 2016, as an increasing number of overdose deaths were attributable to fentanyl, the highly potent synthetic opioid, the US Drug Enforcement Administration issued misinformation about fentanyl exposure. It warned that “fentanyl can be absorbed through the skin or accidental inhalation of airborne powder can also occur …. Just touching fentanyl or accidentally inhaling the substance during enforcement activity or field testing the substance can result in absorption through the skin …. The onset of adverse health effects, such as disorientation, coughing, sedation, respiratory distress or cardiac arrest is very rapid and profound, usually occurring within minutes of exposure” (DEA, 2016). The statement was accompanied by a video, and both were disseminated to law enforcement officials across the nation through channels such as the United States Department of Justice (USDOJ, 2017) the National Police Foundation (NPF, 2016), and a similar warning was released by the Center For Disease Control and Prevention (CDC, 2019).

Given the perceived credibility of these sources, this misinformation propagated widely across mainstream and social media, as well as through policing informational networks (Attaway et al., 2021; Beletsky et al., 2020). It may lead officers to take unnecessary precautions in responding to scenes where fentanyl is suspected, wasting time in effective overdose response. It also perpetuates community-wide stigma against people who use drugs by inaccurately portraying them as toxic and dangerous to be around. The misinformation may further aggravate stress and burnout among police by placing an extraordinary but unfounded mental strain on police officers, perpetuating the belief that they can quickly die from touching or breathing a substance police routinely encounter. Indeed, many of the reported fentanyl exposure incidents among police share the symptoms of a panic attack rather than an opioid; overdose, and no incidents to our knowledge have been confirmed as overdoses by exposure (Herman et al., 2020).

It is critical to correct these false beliefs for the benefit of both occupational and public health. To that end, he American College of Medical Toxicology and the American Academy of Clinical Toxicology issued a joint report in 2017 asserting the risk of fentanyl overdose via incidental transdermal exposure is very low, and it would take 200 min of breathing fentanyl at the highest airborne concentrations to yield a therapeutic dose, but not a potentially fatal one (Moss et al., 2018). In the very unlikely event of a substantial exposure by either means, the slow onset of opioid toxicity would allow sufficient time to detect and reverse it (Moss et al., 2018). Regardless, police officers nationwide continue to report uncorroborated cases of overdose by dermal or inhalation exposure to fentanyl while social and mainstream media spread these reports without verification, with the social media posts reaching 70 million viewers or more (Beletsky et al., 2020). Most recently, the San Diego Sheriff released a dramatic body camera video of a deputy abruptly falling over after testing recovered fentanyl in the field, alleging he only survived because he was quickly given four doses of intranasal naloxone, and warning viewers they too could quickly overdose from casual or incidental contact with trace amounts of fentanyl (Saunders, 2021). The video was widely reported by national media outlets and received millions of views before a group of interdisciplinary experts decisively rebutted the claim that the event was a fentanyl overdose (Figueroa & Kucher, 2021). This highlights the urgent need for corrective messaging (Attaway et al., 2021).

Despite a consistent stream of media accounts of overdose from fentanyl exposure, few studies have reported on officer perceptions of this misinformation. A survey of first responders in New York found that 80% believed “briefly touching fentanyl could be deadly” (Persaud & Jennings, 2020). To our knowledge, only one study has reported on the effects of correcting this perception. A brief training intervention conveyed accurate information about fentanyl exposure to 113 officers in Missouri and greatly increased respondents’ ability to identify the statement “I can overdose from touching fentanyl” as false, a statistically significant change from 20.9% to 83.6% (Winograd et al., 2020). To confirm the potential of such corrective messaging, this study examined the effects of a similar training intervention among Indiana police officers.

## Methods

SHIELD (Safety and Health Integration in the Enforcement of Laws on Drugs) is a training program for law enforcement officials about harm reduction and occupational safety (SHIELD, 2021). It delivers information about needle stick injuries, officer mental health, the public safety benefits of harm reduction programs and addiction treatment and a 10-min module about fentanyl exposure. Three 3-h SHIELD training sessions were conducted in Indiana in December 2020, and March and June 2021. The trainings were held via Zoom to limit the spread of COVID-19, and were led by a former police officer to maximize credibility and present material in ways that would resonate with officers.

The module emphasized that a nicotine transdermal patch is specially formulated to promote the absorption of nicotine through the skin. This effect cannot be achieved by exposing skin to tobacco, and the same principle applies to fentanyl. The training stated that people routinely process and package illicit fentanyl in large quantities in confined spaces and there are no reports of transdermal or inhalation overdoses among this group, nor any evidence of them taking precautions while around fentanyl. Finally, it emphasized that false beliefs about fentanyl’s lethality can produce physical reactions consistent with severe panic, but that these symptoms are highly inconsistent with, if not contradictory to, those of exposure to fentanyl.

The study was approved exempt by the Wayne State University IRB as a training evaluation with minimal risk (#2020037). Using IBM SPSS Statistics (IBM Corp., 2020), we conducted descriptive statistics on all variables and completed bivariate analyses (χ2, ANOVA, independent and paired *t*-tests) to assess for attitudinal differences by respondent characteristics and to assess change before and after the training to the survey item “First responders who encounter fentanyl are at great risk of overdose by touching it or inhaling it” which was scored on a 6-point Likert scale (1 = strongly agree, 2 = agree, 3 = somewhat agree, 4 = somewhat disagree, 5 = disagree, and 6 = strongly disagree).

## Results

SHIELD trainings are facilitated by the Indiana Law Enforcement Academy and in total, 204 officers from 49 Indiana counties attended. Of these individuals, 129 completed the baseline survey, which was administered immediately prior to training, for a response rate of 63.2%. After the training, participants were asked to immediately complete the follow-up survey; of the 129, 53.5% (*n* = 69) did so, and could be linked to their pre-survey using a code derived from name and date of birth. As illustrated in Table 1, respondents who completed the baseline and follow-up surveys were predominantly white males, of various ranks and service histories, with an average age of 45. Importantly, over half of respondents (61.2%; *n* = 79) reported that they had previously received “fentanyl exposure precaution” training, i.e., the type of misinformation the SHIELD module seeks to correct.

**Table 1:**
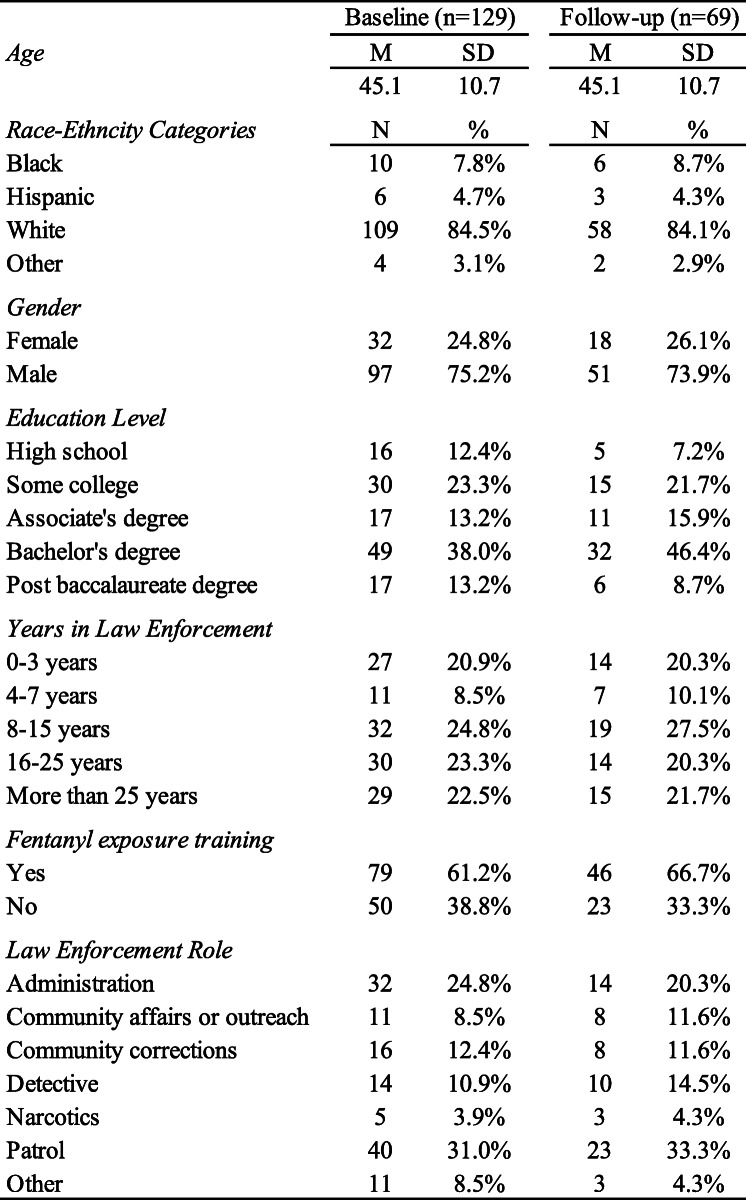
Respondent Characteristics at Baseline and Follow-up

Officers were asked to rate how much they agree with the statement “First responders who encounter fentanyl are at great risk of overdose by touching it or inhaling it” and as illustrated in Table 2, baseline survey results (*N* = 129) suggest that 79.8% (*n* = 103) agreed (strongly agree, agreed, somewhat agree) that there is risk of overdose from touching or inhaling fentanyl while 20.1% (*n* = 26) disagreed (strongly disagree, disagree, somewhat disagree). We examined differences in responses by officer characteristics and found that that those who earned a college degree (*n* = 83) were significantly less likely to perceive risk from fentanyl than those who did not (2.5 and 1.9 respectively; *t* = 2.17, 95% CI [1.14, 0.05], Cohen’s d = 2.33, *p* = .032). Similarly, there were significant differences by law enforcement role (F = 2.71, *p* = .017) in that patrol officers were significantly more likely to perceive fentanyl risk than others (2.6 and 1.8 respectively; *t* = 2.95, 95% CI [0.26, 1.32], Cohen’s d = 1.61, *p* = .004); however, there were no differences by age, race, gender, length of service, or prior fentanyl-related training.

**Table 2:**
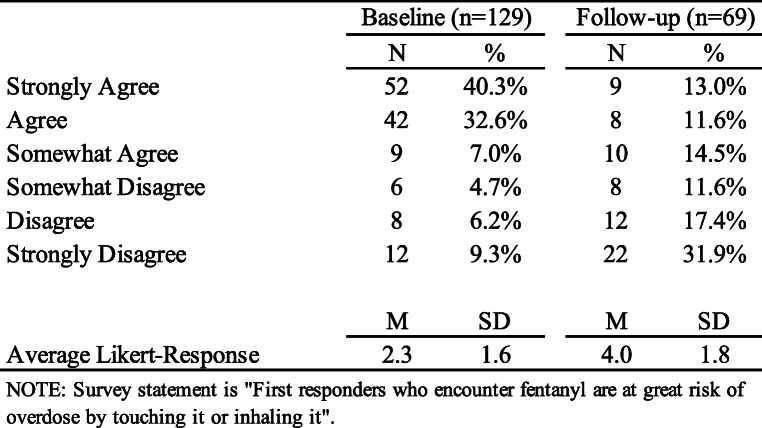
Fentanyl Survey item Responses at Baseline and Follow-up

Among those who completed the post training follow-up survey (*n* = 69), responses changed from 79.8% (*n* = 103) agreeing with the fentanyl exposure misinformation statement to 39.1% (*n* = 27 of 69) after training with 60.9% (*n* = 42) disagreeing. Paired t-tests revealed a statistically significant change in pre- and post-test responses to this item (2.2 to 4.0; *t* = 6.52, 95% CI [1.05, 0.51], Cohen’s d = 2.33, *p* < .001). Moreover, there were no longer significant differences by education, officer role, or any of additional respondent characteristics.

## Discussion

Building on the findings of the Missouri study, this analysis confirms that a brief training module about the minimal dangers of fentanyl exposure had a corrective effect on police officers’ belief that they are at great risk of overdose from inhaling or touching it. In both cases, the intervention had a considerable effect on the measured outcome. This suggests a training video with similar content administered to police officers at roll calls or other institutional settings holds promise, especially given the ease by which such a product could be widely disseminated over the Internet.

There are limitations to this study. The overall post-training response rate of 34% can be considered low as compared to the baseline rate of 63%, and 69 respondents is a small number that can suggest limited confidence in the conclusions. This attrition and the overall response rate are not necessarily indicative of a nonresponse bias, however. Response rates to online surveys administered in unsupervised settings are frequently within this range, and police survey research commonly yields such rates (Nix et al., 2019). While the initial baseline survey was administered during an allotted portion of time at the commencement of training, respondents were asked to complete the follow-up instrument on their own time afterward. Officers may have had work obligations that precluded responding, or lacked convenient access to the internet following the training. Importantly, an analysis of the respondents who completed both the baseline and post-training surveys suggests there were no significant demographic differences compared to those who only completed the baseline. Lastly, these results are meant to confirm the findings of an earlier pilot study regarding what remains a novel intervention. The considerable effect sizes observed in both cases speak to the potential of a rigorously evaluated intervention implemented on a larger scale.

The effects of the module were not sensitive to officer education level, but this study did not account for local differences in police culture. Therefore, it is unclear the extent to which the results can be generalized across police departments. Notably, the intervention tested knowledge, not behavior, so while there is evidence the training corrected misinformation, it is unclear if police behavioral intentions were influenced accordingly. The study also did not examine if its effects persisted or if countervailing misinformation caused variance of beliefs over time. If agency executives and labor union officials were to endorse the training, it is likely its effects would be amplified.

## Conclusion

As jurisdictions contemplate lessening the role of police in response to overdose, addiction-related behaviors will continue to generate police response and place them in contact with people who use drugs (del Pozo et al., 2021). This intervention supplies police officers with accurate information about the risks posed by incidental fentanyl exposure. It can promote a more effective public health response to the overdose crisis, one based on a more accurate assessment of the risks police officers face, especially when beliefs held by police shape the initial response to an incident and influence the beliefs of the constituencies they serve. Given the observed rates of COVID-19 vaccine hesitancy among actively serving police officers and the obstacles this poses for mandates critical to population health (del Pozo & Wood, 2021), it is important to encourage evidence-based decision-making among police not only in cases of exposure to fentanyl, but in evaluating individual and collective threats to health more generally.

## Data Availability

The datasets during and/or analyzed during the current study available from the corresponding author on reasonable request.
